# Quantification of airway wall contrast enhancement on virtual monoenergetic images from spectral computed tomography

**DOI:** 10.1007/s00330-023-09514-2

**Published:** 2023-03-09

**Authors:** Arndt Lukas Bodenberger, Philip Konietzke, Oliver Weinheimer, Willi Linus Wagner, Wolfram Stiller, Tim Frederik Weber, Claus Peter Heussel, Hans-Ulrich Kauczor, Mark Oliver Wielpütz

**Affiliations:** 1 Department of Diagnostic and Interventional Radiology, Heidelberg University Hospital, Im Neuenheimer Feld 420, 69120 Heidelberg, Germany; 2 Translational Lung Research Center Heidelberg (TLRC), German Center for Lung Research (DZL), Im Neuenheimer Feld 156, 69120 Heidelberg, Germany; 3 Department of Diagnostic and Interventional Radiology With Nuclear Medicine, Thoraxklinik at University of Heidelberg, Röntgenstraße 1, 69126 Heidelberg, Germany

**Keywords:** Multidetector computed tomography, Lung, Contrast material, Computer-assisted image processing

## Abstract

**Objectives:**

Quantitative computed tomography (CT) plays an increasingly important role in phenotyping airway diseases. Lung parenchyma and airway inflammation could be quantified by contrast enhancement at CT, but its investigation by multiphasic examinations is limited. We aimed to quantify lung parenchyma and airway wall attenuation in a single contrast-enhanced spectral detector CT acquisition.

**Methods:**

For this cross-sectional retrospective study, 234 lung-healthy patients who underwent spectral CT in four different contrast phases (non-enhanced, pulmonary arterial, systemic arterial, and venous phase) were recruited. Virtual monoenergetic images were reconstructed from 40–160 keV, on which attenuations of segmented lung parenchyma and airway walls combined for 5th–10th subsegmental generations were assessed in Hounsfield Units (HU) by an in-house software. The spectral attenuation curve slope between 40 and 100 keV (*λ*HU) was calculated.

**Results:**

Mean lung density was higher at 40 keV compared to that at 100 keV in all groups (*p* < 0.001). *λ*HU of lung attenuation was significantly higher in the systemic (1.7 HU/keV) and pulmonary arterial phase (1.3 HU/keV) compared to that in the venous phase (0.5 HU/keV) and non-enhanced (0.2 HU/keV) spectral CT (*p* < 0.001). Wall thickness and wall attenuation were higher at 40 keV compared to those at 100 keV for the pulmonary and systemic arterial phase (*p* ≤ 0.001). *λ*HU for wall attenuation was significantly higher in the pulmonary arterial (1.8 HU/keV) and systemic arterial (2.0 HU/keV) compared to that in the venous (0.7 HU/keV) and non-enhanced (0.3 HU/keV) phase (*p* ≤ 0.002).

**Conclusions:**

Spectral CT may quantify lung parenchyma and airway wall enhancement with a single contrast phase acquisition, and may separate arterial and venous enhancement. Further studies are warranted to analyze spectral CT for inflammatory airway diseases.

**Key Points:**

*• Spectral CT may quantify lung parenchyma and airway wall enhancement with a single contrast phase acquisition.*

*• Spectral CT may separate arterial and venous enhancement of lung parenchyma and airway wall.*

*• The contrast enhancement can be quantified by calculating the spectral attenuation curve slope from virtual monoenergetic images.*

**Supplementary Information:**

The online version contains supplementary material available at 10.1007/s00330-023-09514-2.

## Introduction

Quantitative post-processing of computed tomography (CT) is an established method to assess abnormalities of the lung parenchyma and airways in pulmonary fibrosis, cystic fibrosis, asthma, or chronic obstructive pulmonary disease [1–7]. Inflammatory alterations of lung density and airway dimensions can be reversible, in contrast to emphysema and bronchiectasis, for example, after smoking cessation [8, 9]. Thus, inflammation is measured indirectly based on lung density and airway wall dimensions [6–9]. Usually, non-enhanced CT acquisitions are recommended since contrast material alters the results of lung density and airway dimension measurements [10–12]. Lung parenchyma contrast enhancement has little been studied to date with CT, but studies using magnetic resonance imaging (MRI) indicate that parenchymal enhancement may reflect active inflammation [13, 14]. Also, due to pulmonary vessel remodeling in inflammatory airway diseases, contrast enhancement of the airway wall is appreciated in clinical imaging [15, 16]. In MRI, increased gadolinium uptake of the inflamed airway wall is seen in cystic fibrosis for example [17]. Therefore, the assessment of lung parenchyma and airway wall contrast enhancement might be of value for radiologists when characterizing inflammatory airway diseases, but it has not been studied systematically.

The quantification of enhancement with conventional CT would require the comparison of non-enhanced with contrast-enhanced CT acquisitions [18]. Exactly identical acquisition settings, breath hold position, and segmentation or even registration would be required for a precise quantification of lung and airway wall contrast enhancement [19, 20]. Spectral detector CT uses one X-ray tube and two different detector layers to selectively absorb low- and high-energy photons simultaneously from the polychromatic X-ray spectrum [21]. Since opacification by iodine-based contrast materials is dependent on photon energy, iodine maps and virtual monoenergetic images (VMI) can be produced from spectral CT [22, 23]. Iodine maps are beneficial in pulmonary embolism and lung tumor characterization, but have not been tested for the assessment of small structures, e.g., subsegmental airway walls [24]. Therefore, VMI could be used to modulate energy levels and thereby iodine-dependent attenuation for subsequent quantification [22, 23]. The spectral attenuation curve slope (*λ*HU) based on measured CT values in Hounsfield Units (HU) on different monoenergetic display energy levels has been proposed for studying contrast enhancement at monophasic spectral CT [25–27]. As another prerequisite, it was previously demonstrated that by modifications to quantitative algorithms, the effects of intravascular contrast material on airway segmentation can be largely mitigated, thus enabling the comparison of different intensities of contrast enhancement [28]. With the present work, we sought to study lung parenchyma and airway wall attenuation as a function of display energy level. Furthermore, we determined lung and airway wall spectral attenuation curve slopes in the non-enhanced, pulmonary arterial, systemic arterial, and venous phase for the quantification of lung parenchyma and airway wall enhancement in lung-healthy individuals.

## Materials and methods

### Patients

**Fig. 1:**
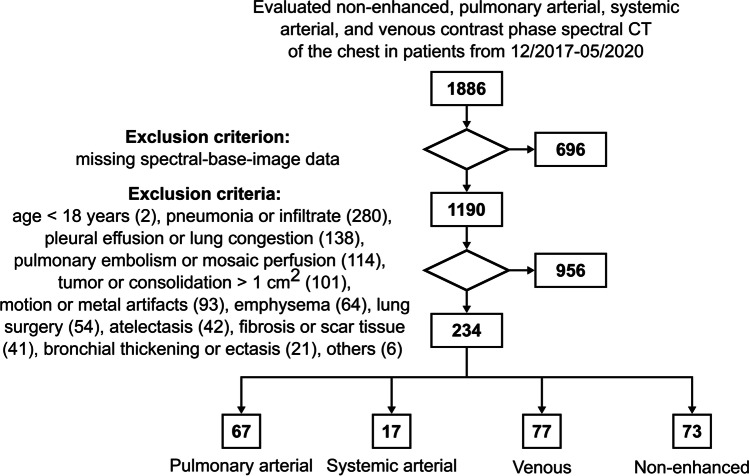
Study flowchart. Number of exclusions are given in brackets. The following exclusion criteria are summarized under others (6): acquisition protocol deviations (3), patient already included in study with another CT scan (2), post-processing error (1)

This retrospective study was approved by the institutional ethics committee (S-924/2019), and requirement of informed consent for data processing was waived. Via database research encompassing 12/2017–05/2020, 1886 patients who underwent clinically indicated non-enhanced (main indication “nodule detection”), pulmonary arterial (“pulmonary embolism”), systemic arterial (“aortic CT angiography”), or venous (“oncologic staging”) phase contrast-enhanced spectral detector CT of the chest were screened for inclusion and exclusion criteria in consensus by two readers with 2 and 7 years of experience in thoracic imaging, respectively (Fig. 1). In total, 234 patients (125 women, 109 men) aged 54 ± 17 years were recruited. A total of 73 patients had non-enhanced and 161 patients had contrast-enhanced spectral CT, of which 67 were pulmonary arterial, 17 systemic arterial, and 77 venous phase acquisitions (Table 1).

**Table 1:** Patient characteristics. Data given as median (interquartile range). Data for height, weight, and BMI (body mass index) were missing for four patients in the non-enhanced, 27 patients in the pulmonary arterial, one patient in the systemic arterial, and one patient in the venous phase

	Non-enhanced	Pulmonary arterial	Systemic arterial	Venous
*n*	73	67	17	77
Age	55 (45–64)	49 (38–66)	66 (52–73)	53 (36–69)
Sex [m/f]	42/31	19/48	10/7	38/39
Height [cm]	172 (167–180)	168 (164–176)	176 (163–180)	171 (163–178)
Weight [kg]	75 (69–92)	85 (65–104)	77 (66–83)	75 (63–86)
BMI [kg/m^2]	26 (23–29)	28 (24–34)	25 (23–28)	25 (22–28)

### Spectral detector CT

Spectral detector CT of the chest was performed using a 64-row dual-layer CT with dose modulation in inspiratory breath hold (IQon Spectral CT, Philips Healthcare). Contrast material (Iohexol 350 mg iodine/mL, Accupaque 350, GE Healthcare; or Iomeprol 400 mg iodine/mL, Iomeron 400, Bracco Imaging) was applied by a power injector following standardized protocols (Table 2). The median volumetric computed tomography dose index (CTDI_vol_) for non-enhanced, pulmonary arterial, and venous phase acquisitions was 3.8, 7.7, and 5.8 mGy, respectively, while systemic arterial phase acquisitions showed a median CTDI_vol_ of 40.4 mGy. The systemic arterial phase images were acquired with a triple-rule-out contrast protocol, requiring higher dose for the investigation of thoracic aorta, pulmonary arteries, and coronary arteries. Reconstructed slice thickness 1.5 mm and increment 0.75 mm were identical for all groups. Conventional reconstructions reflecting 120 kVp and ten VMI were generated from spectral-base image data using software provided by the CT manufacturer (IntelliSpace Portal 11, Philips Healthcare) at 10-keV intervals from 40 to 100 keV and 20-keV intervals from 120 to 160 keV.

**Table 2:** Spectral CT acquisition protocol and reconstruction. Data given as median (interquartile range). Flow rate, volume, and duration data were missing for 30 patients in the pulmonary arterial and one patient in the venous phase. Please note that the systemic arterial phase is acquired with a dual contrast bolus. *DLP*, dose length product; *CTDI*_*vol*_, volumetric computed tomography dose index

	Non-enhanced	Pulmonary arterial	Systemic arterial	Venous
Acquisition				
Collimation [mm]	64 × 0.625	64 × 0.625	64 × 0.625	64 × 0.625
Pitch	1.014	0.984	0.16/0.18	0.984
Rotation time [s]	0.75	0.33	0.27/0.33	0.33
kVp	120	120	120	120
mAs	41 (37–55)	84 (68–127)	446 (346–564)	65 (54–77)
DLP [mGy*cm]	142 (127–181)	285 (235–419)	1525 (1132–1749)	221 (187–273)
CTDI_vol_ [mGy]	3.8 (3.4–5.0)	7.7 (6.2–11.3)	40.4 (33.0–51.0)	5.8 (4.9–7.0)
Contrast				
Trigger region		Pulmonary trunk	Descending aorta	Pulmonary trunk
Threshold [HU]		150	110	150
Delay [s]		6	5	35
Iodine [mg/mL]		350	400	350
Flow rate [mL/s]		3.4 (3.3–3.9)	4.0 (3.6–4.0) + 3.3 (3.3–3.4)	2.9 (2.9–2.9)
Volume [mL]		55.3 (55.1–65.1)	75.7 (75.4–75.8) + 19.5 (19.4–19.7)	50.1 (50.0–50.3)
Duration [s]		16.8 (14.3–19.0)	19.2 (19.1–21.2) + 5.8 (5.8–6.0)	17.1 (17.1–17.2)
Reconstruction				
Kernel	B	B	CB	B
Spectral level	3	3	4	3
Matrix	512 × 512	512 × 512	512 × 512	512 × 512
Slice thickness [mm]	1.5	1.5	1.5	1.5
Increment [mm]	0.75	0.75	0.75	0.75

### Qualitative and quantitative image analysis

In order to measure vascular opacification, mean CT attenuation in HU was measured manually on conventional reconstruction using a circular region-of-interest in the right pulmonary artery and the descending aorta with an area of 2.0 or 1.5 cm^2^ (± 0.05 cm^2^), and the inferior vena cava with an area of 1.5 or 1.0 cm^2^ (± 0.05 cm^2^). The well-validated scientific software YACTA (version 2.9.1.12/16/31) segmented the lungs and the airway tree on all eleven reconstructions as previously described [2, 8–10, 28–32]. Mean lung density (MLD), representing the mean attenuation of all segmented lung voxels, the 15th percentile of the lung density histogram (Perc15), and lung vessel volume (VV), defined as the volume of intrapulmonary segmented blood vessels, were quantified as previously described [9, 10, 31]. The airway tree was analyzed from the 1st (trachea) to the 10th airway generation. The values for the 5th–10th generation (G5–10) were aggregated as a pooled parameter for subsegmental airways to handle excessive data for high airway generations. Aggregated data for subsegmental airway generations was shown to be sensitive to smoking cessation, COPD disease progression, and also bronchial thermoplasty for asthma [8, 30, 32]. The mean and standard deviation (SD) of HU of intra-tracheal air and the number of segmented airway segments were recorded. Airway measurements were calculated using the modified integral-based method which removes airway wall segments attached to a vessel from calculations of all airway parameters, thus avoiding blooming of contrast enhancement into airway measurements, as previously described [28]. The airway dimensions wall thickness (WT), representing the median distance between the inner and the outer airway wall border, and total diameter (TD), defined as the median distance from the outer to the outer border of the airway segment, were quantified as previously described [2, 8, 28, 30, 32]. As a standardized measure of airway wall thickness, the AWT-Pi10 was determined by the square root of the wall area for an airway with an internal perimeter of 10 mm as previously described [33]. Finally, the median maximum airway wall attenuation (MM) in HU was recorded [2, 29, 34.﻿]. For WT, TD, and MM, mean values were computed for the whole lung from all measurements of an airway generation. The approximated *λ*HU was calculated for a line through 40 keV and 100 keV values for MLD and for MM by the previously described formula $\lambda \mathrm{HU }={~}^{{\mathrm{HU}}_{40\mathrm{keV}}-{\mathrm{HU}}_{100\mathrm{keV}}}\!\left/ \!{~}_{60\mathrm{keV}}\right.$, as the spectral curve flattens for higher keV levels [25, 27].

### Statistical analyses

All data were recorded in a dedicated spreadsheet (Excel, Microsoft Corporation), and analyses were performed with SPSS (SPSS Statistics 27, IBM). Data are given as median and interquartile range, or as mean ± standard deviation. Exclusively “complete case analyses” were carried out regarding quantitative CT data. The number of subjects with missing values in the raw data is given in the figure and table legends for each parameter. These subjects were not included in the analysis. Quantitative imaging parameters within contrast phase groups were compared using one-way analysis of variances (ANOVA) for repeated measures or the non-parametric Friedman test. Post hoc analyses were performed using paired *t*-test or Dunn’s test with Bonferroni correction for multiple testing. Quantitative imaging parameters between contrast phase groups were compared with one-way ANOVA and the post hoc Tukey test or with ANOVA on ranks and the post hoc Dunn-Bonferroni test as appropriate, depending on residual distribution. Statistical significance was accepted for *p* < 0.05.

## Results

### Contrast phase and display energy (keV) influence lung densitometry

**Fig. 2:**
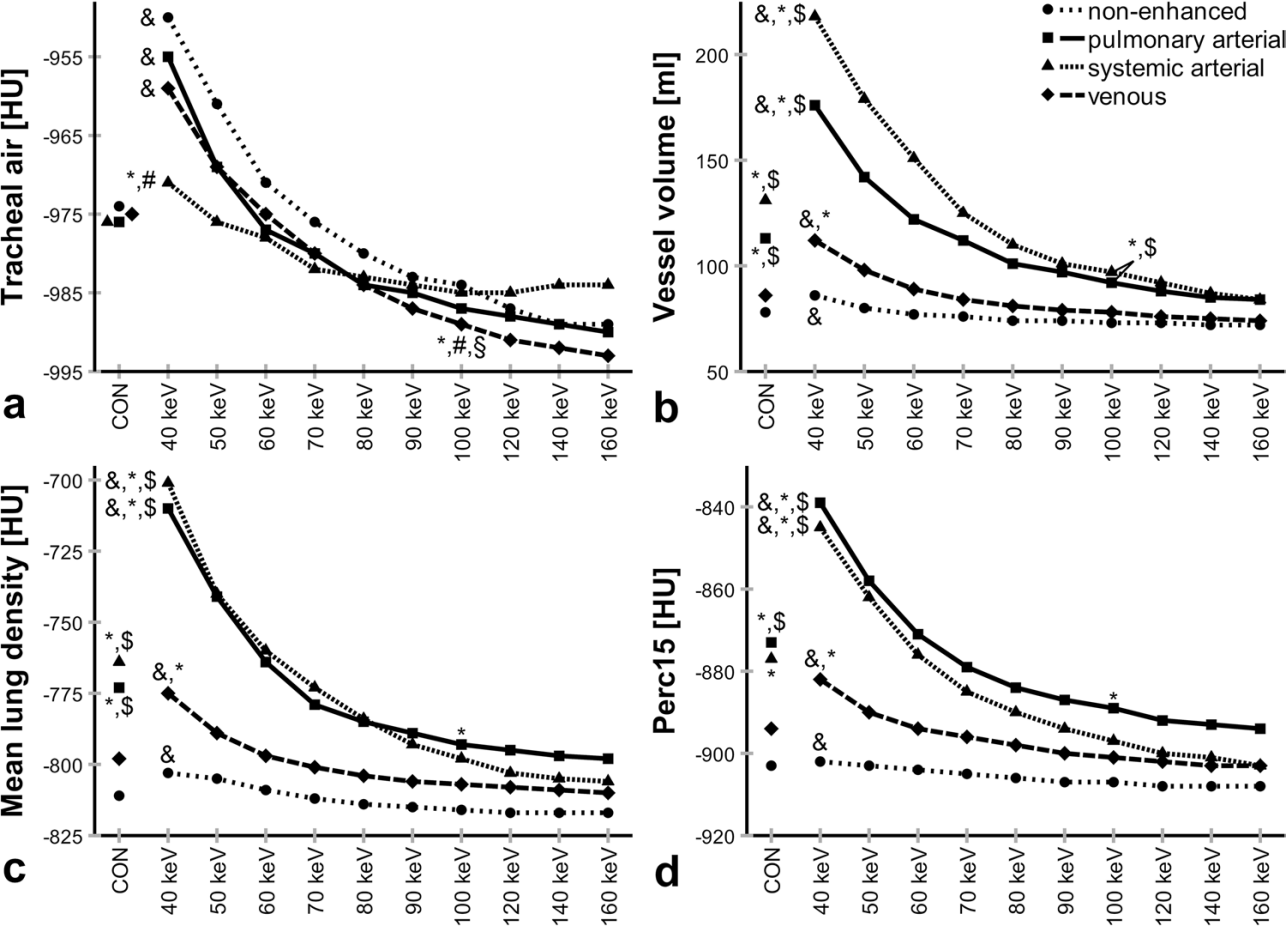
**a**–**d** Influence of contrast phase and display energy (keV) on lung densitometry. CON, conventional; Perc15, 15th percentile of the lung density histogram. Data are given as median. *p < 0.001–0.05 vs. non-enhanced, #*p* < 0.001–0.05 vs. pulmonary arterial, §*p* < 0.001–0.05 vs. systemic arterial, $*p* < 0.001–0.05 vs. venous, &*p* < 0.001–0.05 vs. 100 keV. Kindly note that for six subjects, some datapoints were missing

In order to assess the influence of contrast material on lung densitometry, two factors were varied: a non-enhanced and three different contrast phases were studied in conjunction with a conventional reconstruction and VMI in ten different display energy levels (keV). Opacification of the pulmonary artery in the pulmonary and systemic arterial phase showed a similar pattern, and was higher overall compared to that in the non-enhanced and venous phase on conventional reconstructions (*p* < 0.001) (Table 3). The mean density of tracheal air as an independent internal standard showed no significant differences between the different contrast phases on conventional reconstructions (*p* = 0.70) (Fig. 2). Mean and SD (reflecting noise) of tracheal air were highest at 40 keV, followed by a continuous decrease towards 160 keV in the non-enhanced, pulmonary arterial, and venous phase (40 vs. 100 keV, *p* < 0.001), but less pronounced in the systemic arterial phase (HU *p* = 0.15, SD *p* < 0.001) (Electronic Supplementary Material 1).

**Table 3:** Vessel opacification on conventional reconstructions from spectral CT. Region-of-interest measurements in Hounsfield Units (HU) of the right pulmonary artery, descending aorta, and inferior vena cava for non-enhanced, pulmonary arterial, systemic arterial, and venous phase spectral CT. Data given as median (interquartile range). *p < 0.001 vs. non-enhanced, ^#^*p* < 0.001 vs. pulmonary arterial, ^§^*p* < 0.001 vs. systemic arterial, ^$^*p* < 0.001 vs. venous phase

	Non-enhanced	Pulmonary arterial	Systemic arterial	Venous
Right pulmonary artery [HU]	38.8 (34.3–42.9)^#,§,$^	290.8 (223.9–347.5)*^,$^	332.8 (281.2–416.9)*^,$^	114.6 (102.2–131.3)*^,#,§^
Descending aorta [HU]	43.5 (37.5–49.9)^#,§,$^	219.0 (162.5–257.6)*^,$^	338.0 (292.0–438.1)*^,$^	114.0 (102.9–126.5) *^,#,§^
Inferior vena cava [HU]	38.0 (34.4–41.5)^§,$^	40.9 (37.7–47.7)^§,$^	114.9 (77.7–130.8)*^,#^	90.9 (78.4–108.2)*^,#^

On conventional reconstructions, MLD, VV, and Perc15 were significantly higher in pulmonary and systemic arterial phase compared to those in non-enhanced and venous phase CT (*p* < 0.001–*p* = 0.046). As an exception, Perc15 showed insignificantly higher values in the systemic arterial vs. the venous phase (*p* = 0.94) (Fig. 2). Measurements in the venous phase were mostly similar compared to those on non-enhanced acquisitions (*p* = 0.06–0.20).

MLD, VV, and Perc15 were highest at 40 keV, followed by a continuous decrease to 160 keV in all groups (40 vs. 100 keV, *p* < 0.001). The resulting *λ*HU for MLD was significantly higher in pulmonary and systemic arterial phase CT compared to that in non-enhanced and venous phase CT (*p* < 0.001) (Table 4). Also, *λ*HU for MLD was higher in venous vs. non-enhanced phase CT (*p* < 0.001).

**Table 4:** Spectral attenuation curve slope (*λ*HU). *λ*HU calculated for MLD (mean lung density) and MM (median maximum airway wall attenuation), grouped by airway generation for non-enhanced, pulmonary arterial, systemic arterial, and venous phase spectral CT. Data given as median (interquartile range). *p < 0.001–0.05 vs. non-enhanced, ^#^*p* < 0.001–0.05 vs. pulmonary arterial, ^§^*p* < 0.001–0.05 vs. systemic arterial, ^$^*p* < 0.001–0.05 vs. venous phase. Kindly note that for 44 subjects, some datapoints were missing

	Non-enhanced	Pulmonary arterial	Systemic arterial	Venous
MLD [HU/keV]	0.2 (0.1–0.3)^#,§,$^	1.3 (1.0–1.5) *^,$^	1.7 (1.2–1.8) *^,$^	0.5 (0.5–0.6)*^,#,§^
MM G2 [HU/keV]	0.6 (0.1–1.5)^#,§,$^	2.6 (1.3–3.8)*^,$^	2.7 (1.9–5.4) *^,$^	1.4 (0.6–2.0)*^,#,§^
MM G3 [HU/keV]	0.7 (0.3–1.1)^#,§,$^	2.1 (1.1–3.4) *^,$^	2.5 (1.8–4.4) *^,$^	1.1 (0.7–1.7)*^,#,§^
MM G4 [HU/keV]	0.5 (0.0–1.0)^#,§,$^	1.6 (1.0–2.6) *^,$^	1.9 (1.5–2.7) *^,$^	1.0 (0.5–1.7)*^,#,§^
MM G5–10 [HU/keV]	0.3 (− 0.1 to 0.6)^#,§,$^	1.8 (1.3–2.7) *^,$^	2.0 (1.2–3.2) *^,$^	0.7 (0.2–1.3)*^,#,§^

### Contrast phase and display energy (keV) influence airway dimension measurements

**Fig. 3:**
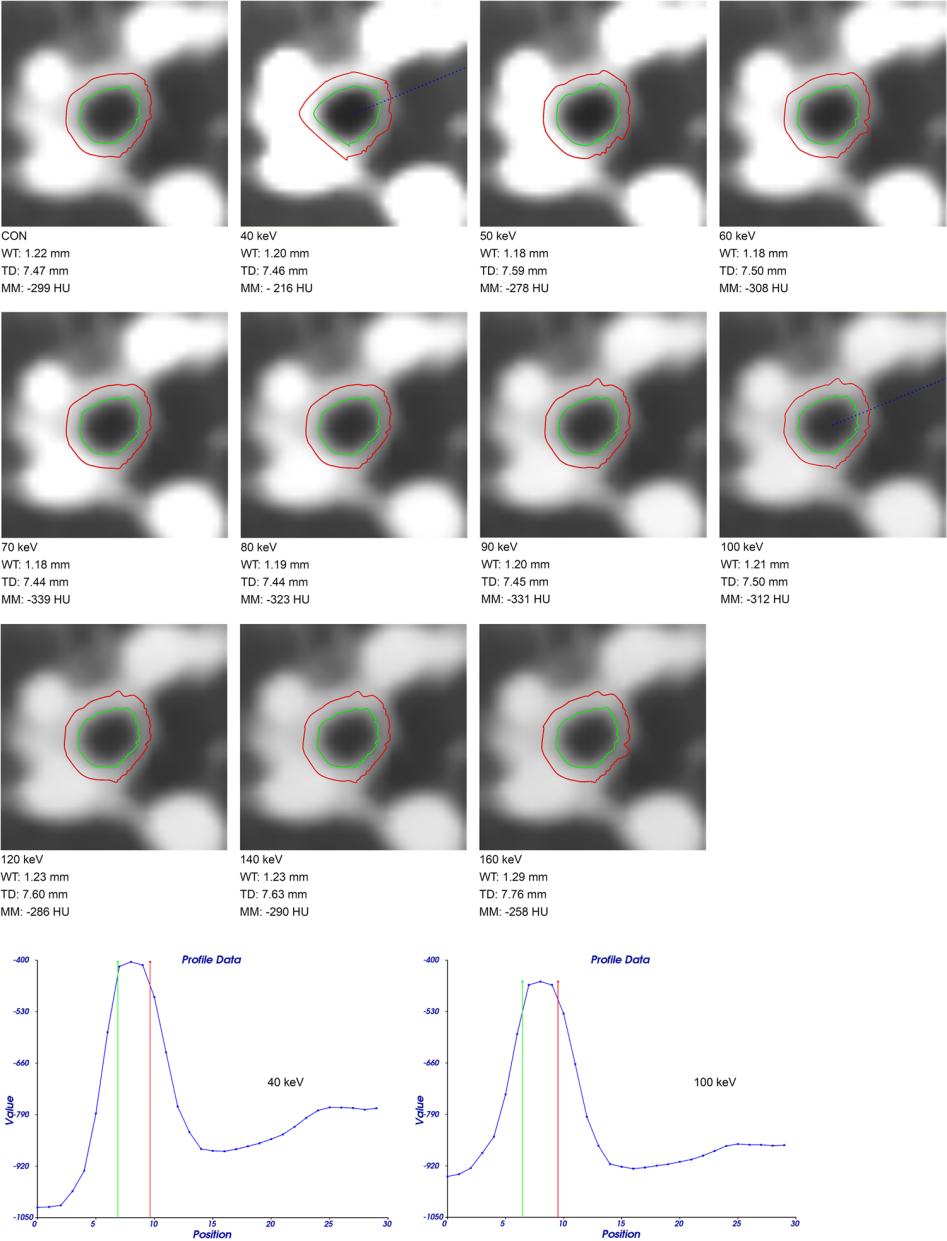
Examples of the segmentation of a subsegmental airway on conventional (CON) reconstruction as well as on virtual monoenergetic images from 40 to 160 keV display energy in pulmonary arterial contrast-enhanced spectral CT. WT, wall thickness; TD, total diameter; MM, median maximum airway wall attenuation. Slices are orthogonal through the airway. Density profiles across the airway wall are shown for 40 and 100 keV, and inner and outer airway wall margins are indicated by green and red lines, respectively. Note that at 40 keV, the airway wall seems to be bulged away from high-contrast vessels. On all reconstructions, these segments are not considered for calculation of airway wall thickness and density etc.

**Fig. 4:**
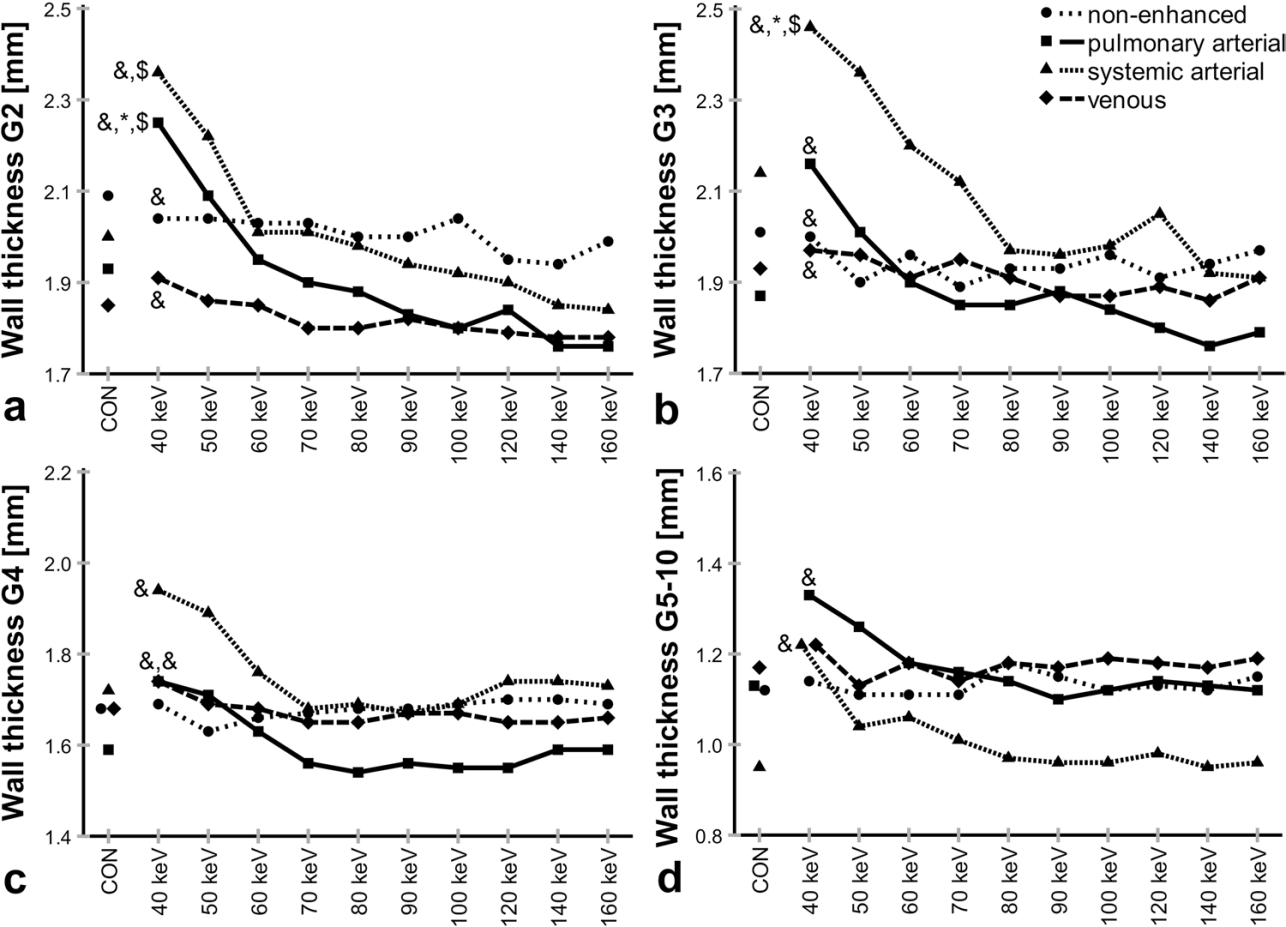
**a**–**d** Influence of contrast phase and display energy (keV) on wall thickness. CON, conventional. Data are given as median. *p < 0.001–0.05 vs. non-enhanced, $*p* < 0.001–0.05 vs. venous, &*p* < 0.001–0.05 vs. 100 keV. Kindly note that for 44 subjects, some datapoints were missing. Also note different *y*-axis scaling for better visualization of subtle differences for distal airway generations

**Fig. 5:**
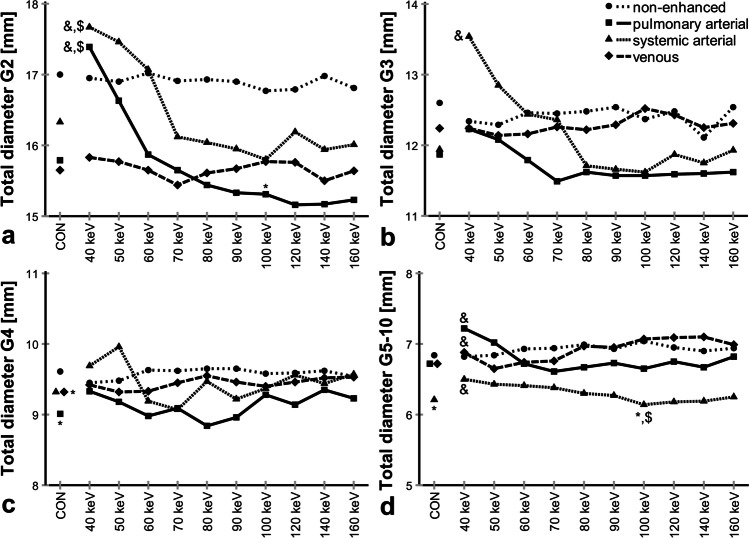
**a**–**d** Influence of contrast phase and display energy (keV) on total diameter. CON, conventional. Data are given as median. *p < 0.001–0.05 vs. non-enhanced, $*p* < 0.001–0.05 vs. venous, &*p* < 0.001–0.05 vs. 100 keV. Kindly note that for 44 subjects, some datapoints were missing. Also note different *y*-axis scaling for better visualization of subtle differences for distal airway generations

Airway dimensions were quantified on the non-enhanced and three different contrast phases on conventional reconstruction and ten VMI to test whether airway wall segmentation is robust against adjacent vessel enhancement and whether airway wall attenuation influences airway dimensions (Figs. 3, 4, and 5). The number of segmented airways was higher on systemic arterial phase images compared to the other groups, but this difference was not statistically significant at any keV level (*p* = 0.21–0.39). The graphs of the other groups were close to each other and showed a slight decrease towards higher energy levels in the non-enhanced and venous phase (40 vs. 100 keV, *p* < 0.001) (Electronic Supplementary Material 2).

On conventional reconstructions, AWT-Pi10 showed no significant difference between contrast phase groups (*p* = 0.31). On VMI, AWT-Pi10 did not differ between the pulmonary arterial and venous phase at any keV level (*p* = 0.06–0.86). Compared to these two contrast phases, the non-enhanced and systemic arterial phase tended to lower values, with the only significant difference being between the non-enhanced and pulmonary arterial phase at 40 keV (*p* = 0.03). Furthermore, AWT-Pi10 was significantly higher at 40 keV compared to 100 keV in both the pulmonary arterial and venous phase (*p* = 0.002–0.003) (Electronic Supplementary Material 3).

On conventional reconstructions, WT showed no significant difference between contrast phase groups (*p* = 0.05–0.93). On VMI, WT showed highest peaks at 40 keV in both arterial phases for all airway generations (40 vs. 100 keV, *p* < 0.001–*p* = 0.004). The non-enhanced and venous phase showed less influence of keV, and no significant influence for subsegmental airways G5–10 (*p* = 0.49–*p* > 0.99). Also, minor differences in WT between contrast phase groups were insignificant for subsegmental airways G5–10 at any keV level (*p* = 0.05–0.81).

On conventional reconstructions, TD showed no significant difference between contrast phase groups for airway generations G2 and G3 (*p* = 0.06–*p* > 0.99), and significant differences between the non-enhanced and venous as well as pulmonary arterial phase for G4 (*p* = 0.001–0.04), and between the non-enhanced and systemic arterial phase for G5–10 (*p* = 0.01). On VMI, TD was significantly higher at 40 keV compared to that at 100 keV in both arterial phases for G2, G3, and G5–10 (*p* < 0.001–*p* = 0.02), but less pronounced in pulmonary arterial phase G3 (*p* = 0.17). In the non-enhanced and in the venous phase, the influence of keV seemed to be small (*p* = 0.21–*p* > 0.99). As an exception, TD showed a significant increase between 40 and 100 keV in the venous phase for G5–10 (*p* = 0.005), relating to the decrease in number of segmented airways on high-energy reconstructions in the venous phase.

### Contrast phase and display energy (keV) influence airway wall attenuation

**Fig. 6:**
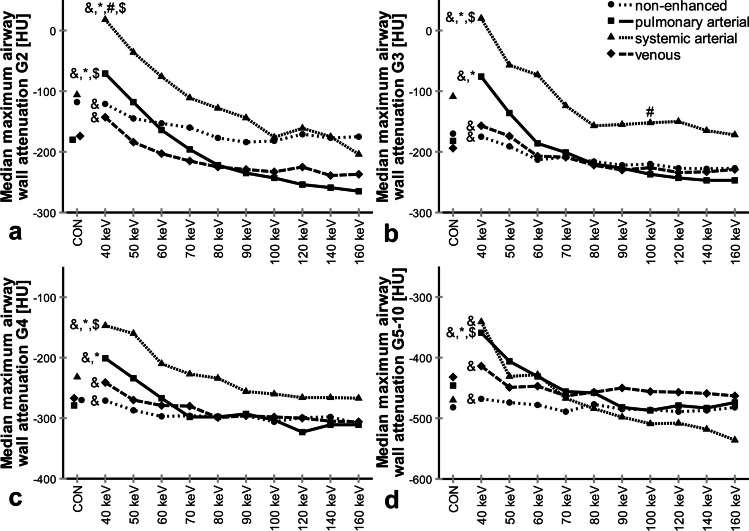
**a–d** Influence of contrast phase and display energy (keV) on median maximum airway wall attenuation. CON, conventional. Data are given as median. *p < 0.001–0.05 vs. non-enhanced, #*p* < 0.001–0.05 vs. pulmonary arterial, $*p* < 0.001–0.05 vs. venous, &*p* < 0.001–0.05 vs. 100 keV. Kindly note that for 44 subjects, some datapoints were missing. Also note different *y*-axis scaling for better visualization of subtle differences for distal airway generations

Next, we measured MM dependence on the contrast phase and keV (Fig. 6). On conventional reconstructions, minor differences between contrast phase groups were insignificant for all airway generations (*p* = 0.07–*p* > 0.99). On VMI, MM peaked at 40 keV, followed by a continuous decrease to 160 keV for all airway generations and contrast phase groups (40 vs. 100 keV, *p* < 0.001–*p* = 0.009). Compared to non-enhanced acquisitions, the peaks at 40 keV display energy were significantly higher in both arterial phases for airway generations G2, G3, and G4 (*p* < 0.001–*p* = 0.003) and only in the pulmonary arterial phase for G5–10 (*p* < 0.001). MM measurements in the non-enhanced and venous phase were similar for all keV (*p* = 0.05–*p* > 0.99).

### Spectral attenuation curve slope quantifies airway wall enhancement

In order to study whether spectral CT can quantify airway wall enhancement based on VMI from one contrast phase, we calculated *λ*HU for MM (Table 4). Slopes from the pulmonary arterial and systemic arterial phase were similar (*p* > 0.99), while both phases showed significantly higher slopes than venous phase and non-enhanced phase images for all airway generations (*p* < 0.001–*p* = 0.03). Also, the venous phase revealed higher *λ*HU than the non-enhanced acquisitions (*p* < 0.001–*p* = 0.03).

## Discussion

With this study, we demonstrate for the first time that monophasic contrast-enhanced spectral detector CT quantifies lung parenchyma and airway wall enhancement based on the spectral attenuation curve slope based on virtual monoenergetic images. Lung densitometry relies on CT numbers in HU for the quantification of emphysema, air-trapping, and fibrosis [4, 6, 35]. With the present work, we show by using conventional reconstructions of dual-layer spectral CT that contrast significantly influences mean lung density. This in turn confirms previous work stating that non-contrast and contrast-enhanced CT are not comparable with regard to densitometry [10, 12]. Furthermore, we show a significant influence of display energy levels of VMI on MLD, VV, and Perc15 with the highest peak on 40-keV images, followed by a continuous decrease towards 160 keV. This effect was stronger in contrast-enhanced images for both arterial phases compared to the venous phase and the non-enhanced acquisition. The curve progression can be explained by the fact that iodine radiation absorption increases towards the k-edge of 33 keV [21, 23]. Since pathology indices such as emphysema index are derived from densitometry, it is conceivable that these are also influenced by contrast phase and display energy level [4, 10, 33, 36]. These findings complement data from Jungblut et al who showed significant influence of intravenous contrast and display energy level on emphysema quantification with decreased emphysema measurements at low keV levels [36]. In this study, the spectral attenuation curve slope for MLD showed steeper slopes in all contrast-enhanced compared to non-enhanced acquisitions (*p* < 0.001), and also steeper slopes in both arterial phases compared to the venous phase (*p* < 0.001). In future studies, a voxel-wise analysis of *λ*HU could be used as a simplified measure to study regional enhancement in inflammatory lung diseases. Of note, this technique provides an alternative to lung iodine maps as already routinely generated from spectral CT, but which have not yet been tested for the assessment of thin airway walls [24, 37].

Airway dimensions have long been studied with quantitative CT as a surrogate for airway disease severity [2, 4, 8, 30]. However, airway wall thickening alone can represent multiple histological changes as edema and active inflammation, as well as chronic remodeling, or reflects inseparable thin layers of mucus [2, 7, 33]. For the present study, a modified integral-based method was employed in order to reduce the influence of contrast media of adjacent vessels on airway wall measurements by excluding affected segments [28]. WT and AWT-Pi10 showed minor trends but did not differ significantly between study groups on conventional reconstruction (WT *p* = 0.05–0.93, AWT-Pi10 *p* = 0.31). However, WT showed the highest peaks at 40 keV in both arterial phases for all airway generations (40 vs. 100 keV, *p* < 0.001–*p* = 0.004). AWT-Pi10 showed peaks at 40 keV in the pulmonary arterial and venous phase (40 vs. 100 keV, *p* = 0.002–0.003). These results demonstrate that the modified integral-based method makes airway dimension measurements more comparable. However, as airway dimension measurements depend on the CT number profile through the airway wall, the increased wall attenuation at low display energies and especially in arterial contrast phases also increased measured wall thickness.

The median maximum airway wall attenuation denotes the actual maximum density in the center of the airway wall and is thus little influenced by variations in the segmentation of the airway wall circumference. It was highest at 40 keV and decreased from 40 to 160 keV display energy in all contrast and non-enhanced phases [21, 23]. Moreover, the peaks at 40 keV were highest in the systemic and pulmonary arterial phase compared to venous and non-enhanced CT. Both findings show that this is an iodine-dependent effect. Previously, *λ*HU was used to measure iodine uptake of lung cancer in correlation with dual-input perfusion CT [26] and to differentiate between histological lung cancer groups [27]. We now show that *λ*HU of the median maximum airway wall attenuation can differentiate arterial from venous and non-enhanced CT for central to subsegmental airway generations (*p* < 0.001–*p* = 0.03), which is more sensitive than using WT as a surrogate for contrast enhancement. Especially the differentiation between arterial and venous enhancement shows the method’s potential to detect changes in contrast material concentration in the airway wall. This enables the evaluation of *λ*HU for its use in inflammatory airway diseases.

Another alternative for the quantification of contrast enhancement regarding lung lesions are iodine density maps derived from spectral CT, which play also a role in pulmonary embolism and thoracic oncologic imaging [24]. However, iodine density maps have not yet been tested for the segmentation of small structures, e.g., intrapulmonary airway walls. Therefore, *λ*HU derived from VMI appeals as a feasible measure of airway wall contrast enhancement. It poses a promising tool for the quantification of airway inflammation and could be integrated into existing post-processing software.

One limitation is the potential for a selection bias, since different patient groups were included in this retrospective study, with different characteristics that might have influenced quantification results. However, anthropomorphic data showed only minor differences between groups (Table 1). For example, the pulmonary arterial phase group showed a different sex ratio and a little higher weight, which might have biased the between-group comparison. But, due to dose considerations a prospective study design with multiple contrast-enhanced scans on the same patient does not seem to be reasonable. As another limitation, reconstruction was performed using the B kernel for non-enhanced, venous, and pulmonary arterial images and the CB kernel for systemic arterial phase images. The kernel was already included in the spectral-base image data and could not be altered retrospectively. Moreover, it should be noted that systemic arterial phase examinations received dual contrast bolus, higher iodine dose of 400 mg iodine/mL, and significantly higher radiation dose, improving conditions for segmentation of distal airways and affecting lung density and airway dimension measurements [11, 19, 38]. However, in this study the notable smaller systemic arterial phase group (*n* = 17) served as a complement for the pulmonary arterial phase images. With this second arterial phase group, we could check whether our findings, especially regarding the comparison between venous and arterial enhancement, were consistent. Compared to the pulmonary arterial phase, the systemic arterial phase did not yield substantially different results.

In summary, we show that virtual monoenergetic images derived from contrast-enhanced spectral CT can be used to regionally quantify lung parenchyma and airway wall enhancement within a single phase acquisition in lung-healthy individuals by calculation of the spectral attenuation curve slope. The method may separate arterial and venous enhancement of the lung parenchyma and airway wall. Further research should evaluate spectral CT for inflammatory airway diseases.

## Supplementary Information

Below is the link to the electronic supplementary material.

Supplementary file1 (PDF 190 KB)

## Abbreviations

CON: Conventional
CTDI_vol_: Volumetric computed tomography dose index
DLP: Dose length product
MLD: Mean lung density
MM: Median maximum airway wall attenuation
Perc15: 15Th percentile of the lung density histogram
TD: Total diameter
VMI: Virtual monoenergetic images
VV: Vessel volume
WT: Wall thickness
λHU: Spectral attenuation curve slope

## Declarations

### Guarantor

The scientific guarantor of this publication is Prof. Dr. Med. Mark O. Wielpütz.

### Statistics and biometry

One of the authors has significant statistical expertise.

### Methodology

*•* retrospective

• cross-sectional study

• performed at one institution
