# Supplementary material for: Quantification of airway wall contrast enhancement on virtual monoenergetic images from spectral computed tomography
Source: Eur Radiol. 2023 Mar 9;33(8):5557–67. doi: 10.1007/s00330-023-09514-2 (PMC10326154; doi:10.1007/s00330-023-09514-2)
Supplement: Supplementary file 1 — Supplementary file1 (PDF 190 KB) [file 330_2023_9514_MOESM1_ESM.pdf]

Electronic Supplementary Material 1

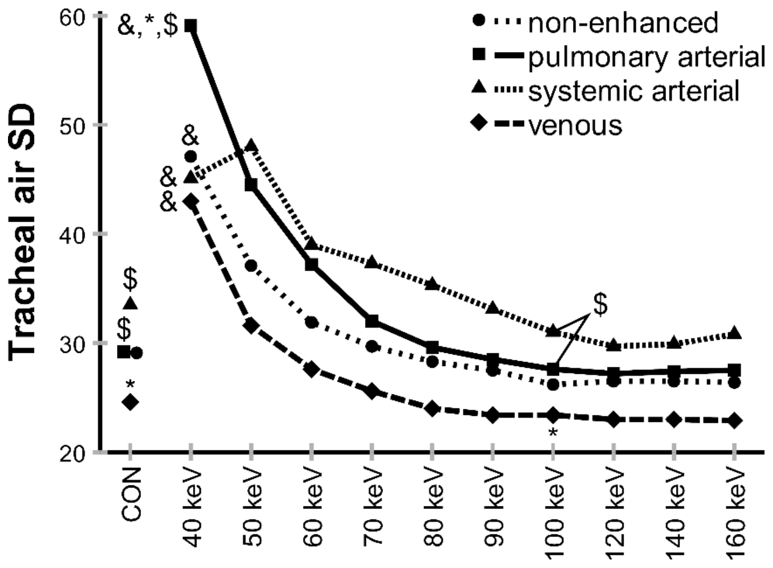

Supplemental Figure S1. Influence of contrast phase and display energy (keV) on noise. Standard deviation (SD) of tracheal air given as median. CON = conventional. * p<0.001-0.05 vs. non-enhanced, $ p<0.001-0.05 vs. venous, & p<0.001-0.05 vs. 100 keV. Kindly note that for one subject, some datapoints were missing.

Electronic Supplementary Material 2

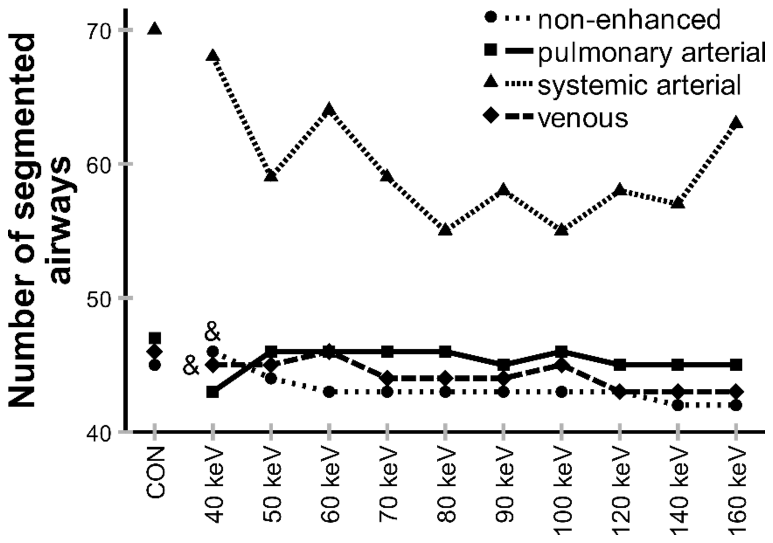

Supplemental Figure S2. Influence of contrast phase and display energy (keV) on the number of segmented airways. Data are given as median. CON = conventional. & p<0.001-0.05 vs. 100 keV. Kindly note that for seven subjects, some datapoints were missing.

Electronic Supplementary Material 3

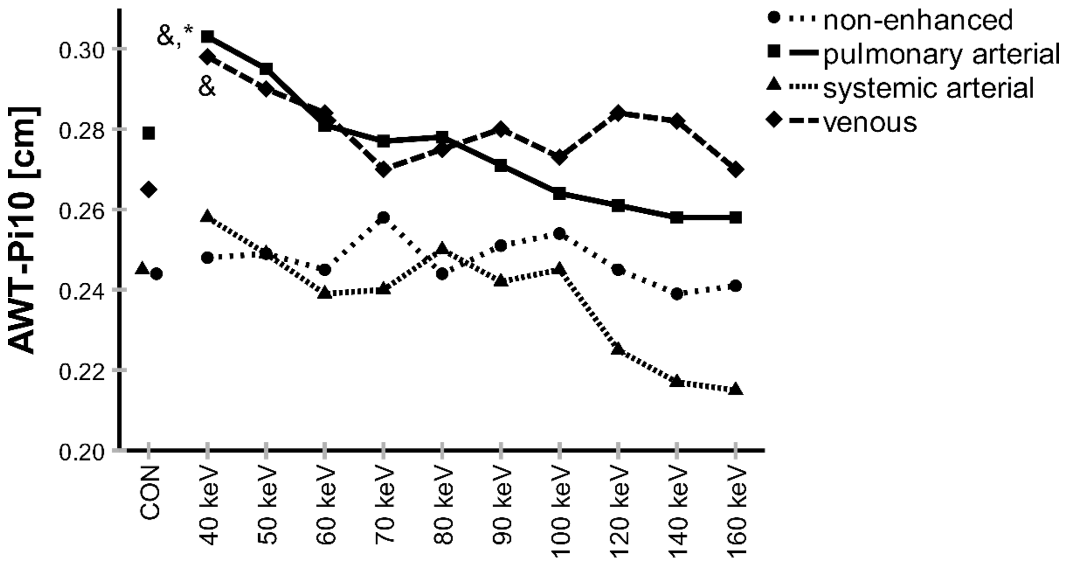

Supplemental Figure S3. Influence of contrast phase and display energy (keV) on AWT-Pi10. The parameter describes a standardized measure of airway wall thickness (AWT) for an airway with an internal perimeter of 10 mm (Pi10) [33]. Data are given as median. CON = conventional. * p<0.001-0.05 vs. non-enhanced, & p<0.001-0.05 vs. 100 keV. Kindly note that for 17 subjects, some datapoints were missing.
